# Genome-Wide Association Studies Reveal Candidate Genes Related to Stem Diameter in Cucumber (*Cucumis sativus* L.)

**DOI:** 10.3390/genes13061095

**Published:** 2022-06-19

**Authors:** Yingying Yang, Shaoyun Dong, Han Miao, Xiaoping Liu, Zhuonan Dai, Xiangsheng Li, Xingfang Gu, Shengping Zhang

**Affiliations:** Institute of Vegetables and Flowers, Chinese Academy of Agricultural Sciences, Beijing 100081, China; yangyingy0801@163.com (Y.Y.); dongshaoyun@caas.cn (S.D.); miaohan@caas.cn (H.M.); liuxiaoping@caas.cn (X.L.); dzhuonan@163.com (Z.D.); 18345967811@163.com (X.L.); guxingfang@caas.cn (X.G.)

**Keywords:** cucumber core germplasm, stem diameter, genome-wide association study, candidate gene

## Abstract

The stem diameter, an important agronomic trait, affects cucumber growth and yield. However, no genes responsible for cucumber stem diameter have been identified yet. In this study, the stem diameter of 88 cucumber core germplasms were measured in spring 2020, autumn 2020 and autumn 2021, and a genome-wide association study (GWAS) was carried out based on the gene sequence and stem diameter of core germplasms. A total of eight loci (*gSD1.1*, *gSD2.1*, *gSD3.1*, *gSD3.2*, *gSD4.1*, *gSD5.1*, *gSD5.2*, and *gSD6.1*) significantly associated with cucumber stem diameter were detected. Of these, five loci (*gSD1.1*, *gSD2.1*, *gSD3.1*, *gSD5.2*, and *gSD6.1*) were repeatedly detected in two or more seasons and were considered as robust and reliable loci. Based on the linkage disequilibrium sequences of the associated SNP loci, 37 genes were selected. By further investigating the five loci via analyzing *Arabidopsis* homologous genes and gene haplotypes, five genes (*CsaV3_1G028310*, *CsaV3_2G006960*, *CsaV3_3G009560*, *CsaV3_5G031320*, and *CsaV3_6G031260*) showed variations in amino acid sequence between thick stem lines and thin stem lines. Expression pattern analyses of these genes also showed a significant difference between thick stem and thin stem lines. This study laid the foundation for gene cloning and molecular mechanism study of cucumber stem development.

## 1. Introduction

Cucumber (*Cucumis sativus* L.), an important vegetable crop, is an annual climbing plant belonging to Cucurbitaceae. In 2020, cucumber was grown on 2,261,318 hectares with a total production of 91,258,272 tons worldwide [1]. With the reduction in cultivated land area and the increase in the demand for fresh vegetables, efficient utilization of limited resources has become essential in cucumber production. The stem, an important part of the cucumber plant, plays a role in support and nutrient transport [2]. Yang [3] analyzed the combining ability of stem diameter of mini cucumber in the greenhouse and found that stem diameter showed a positive effect on yield. Cao [4] treated cucumber plants with different LED spectra and found that blue light could significantly increase the stem diameter of cucumbers. In addition, the application of biological fertilizer has a positive effect on the diameter of the plant stem, and the yield-related traits [5]. Kamil [6] found that the growth index of stem diameter was positively correlated with yield. In sum, thick-stemmed cucumber plants are important for increasing yields when available arable land is limited. Identifying the key genes and molecular mechanisms that control stem structure is critical for effectively modifying plant with desired structural characteristics.

Studies on the stem diameter of cucurbitaceae crops focus on genetic analysis and QTL mapping, and no functional genes have been cloned yet. Qi [7] constructed a four-generation population using muskmelon and cantaloupe as parents, and clarified that the melon stem diameter was inherited by two pairs of additive-dominant-epistatic major genes and additive-dominant polygenic genes. Rao [8] conducted a genetic analysis of the stem diameter of bitter gourd, and found that the inheritance of stem diameter conformed to the additive-dominant-epistatic polygenic genetic model. Lin [9] constructed a linkage group of chieh-qua, and detected one QTL of the chieh-qua stem diameter on LG2, with the phenotypic variation rate of 10.3%. At present, there are few studies on the inheritance and gene mapping of stem diameter in cucumber. Li [10] used the F_2:3_ populations constructed by S94 (North China type) and S06 (European greenhouse type) as parents to map the QTLs of the cucumber stem diameter, and detected two loci—*qMSD-1* and *qMSD-7,* with a heritability of 8.5%. It is difficult to develop molecular markers closely linked with stem diameter that can be used for molecular-assisted breeding.

In recent years, with the development of sequencing technology and the low sequencing costs, genome-wide association study (GWAS) has been widely used in identifying genes’ underlying important agronomic traits in many species [11,12]. In terms of cucumber fruit quality traits, Shang [13] detected the bitterness gene *Bi* in 115 cucumber germplasm resources by GWAS. Bo [14,15] found a new locus *fsd6.1* associated with spines and two loci, *qgf5.1* and *qgf3.1*, related to green fruit flesh in cucumber. For cucumber disease-resistance traits, 395 cucumber germplasm resources were used to analyze downy mildew resistance, and 18 loci were related to downy mildew resistance, five candidate genes were predicted at the major effect loci [16,17,18]. Jia [19] used 231 cucumber core germplasm (CG) resources around the world for powdery mildew resistance analysis, detected 12 loci associated with powdery mildew resistance, and predicted 63 candidate genes. For cucumber abiotic stress resistance, Wang [20] detected four loci related to low-temperature resistance in seedlings, Zhang [21] detected five loci associated with high-temperature resistance in seeds, and Wei [22] detected seven loci related to high-temperature resistance in seedlings and predicted 67 candidate genes at loci *gHII4.1* and *gHII4.2*. Thus far, no research on identifying candidate genes related to the cucumber stem diameter via GWAS has been reported yet.

In this study, the genetic diversity of stem diameter was analyzed using 88 cucumber core germplasms, and GWAS was performed based on the gene sequence and stem diameter of core germplasms. Robust loci that were repeatedly detected in two or more seasons were further investigated to identify candidate genes significantly associated with cucumber stem diameter. The findings can provide a basis for the cloning and molecular mechanism study of cucumber stem diameter-related genes.

## 2. Materials and Methods

### 2.1. Plant Materials

The 88 cucumber CG lines were obtained from the Institute of Vegetables and Flowers, Chinese Academy of Agricultural Sciences, China. These CG lines were chosen from 3342 cucumber resources from all over the world and were thus widely representative [23,24]. All plant materials have been sequenced [24], and the information of ID number, the Individual code, the Group, and Geographic Origin are shown in Table S1.

### 2.2. Phenotypic Data Collection and Analysis

The 88 CG lines were phenotyped three times in different seasons and environments, namely 2020S (field in Nankou farm (40°13′ N, 116°09′ E), Spring 2020), 2020A (plastics greenhouses in Nankou farm (40°13′ N, 116°09′ E), Autumn 2020) and 2021A (plastics greenhouses in Shunyi farm (40°15′ N, 116°83′ E)). The experimental design included a completely randomized block design method with three replicates for each experiment and five plants in each replicate. The diameter of the 15th internodes was measured with a vernier caliper 42 days after transplanting.

### 2.3. Genetic Diversity of Stem Diameter in Germplasm

The average stem diameter of CG lines in three seasons was calculated, respectively. Statistical analysis of phenotypic data was performed using SAS v9.3 [25]. The Pearson correlation coefficient for the traits in each population was estimated with the PROC CORR function based on the maximum value of each experiment [26]. To cluster the CG lines based on their stem diameter, a phylogenetic tree was constructed using TBtools v1.069 based on the average stem diameter of each CG line in three seasons and presented in a heatmap [27].

### 2.4. Resequencing Data Analysis and Genotype

The complete genome of the CG population has been sequenced, and the gene sequence data can be found in Qi [24]. Loci with minimum allele frequency <0.05 were filtered out. Only dimorphic variation sites were retained. These SNPs were screened based on the secondary allele frequency being >0.05 and the integrity >0.8. Finally, 53,921 high-quality and locally unique SNPs uniformly distributed on seven chromosomes in cucumber were used for subsequent analysis [24]. Genome-wide LD analysis was performed using the filtered SNP matrix, and the LD coefficients (r2) between two high-quality SNPs were calculated using the Plink software [28].

### 2.5. Genome-wide Association Analysis

According to the SNP data of association analysis reported by Qi [24], GWAS was performed with 88 CG lines using the factored spectrally transformed linear mixed model (FaST-LMM) [29]. The correlation matrix was estimated as a covariable to record the minimum *p* value of the complete genome, and the Manhattan plot was generated by the CMplot R package in R [30].

### 2.6. Linkage Disequilibrium Analysis

Genome-wide linkage disequilibrium (LD) was analyzed with a filtered SNP matrix, and the LD coefficient (r^2^) between two high-quality SNP was calculated using the software Plink. The parameters were set to: ‘-r^2^-ld-window 999999-ld-window-kb 1000-ld-window-r^2^ 0’, and the results were used to estimate the LD decay.

### 2.7. Prediction of Candidate Genes

According to the SNPs obtained from GWAS, the 50-kb SNP flanking regions were considered candidate regions [24]. LD block interval based on population linkage disequilibrium analysis was used to predict candidate genes using the sequencing information on the cucumber genome database website. Gene function was analyzed and predicted by BLAST in the Cucurbit Genomics Database (CuGenDB) [31], the *Arabidopsis* Information Resource (TAIR), and Gene Ontology (GO) databases [32]. Candidate genes were selected based on the gene annotation and the function of their homologs in other plants and the SNP variations.

### 2.8. Candidate Genes Analysis

To identify candidate genes, cucumber thick stem samples (CG9, CG25, CG26, CG39, CG94, CG107) and thin stem samples (CG1, CG14, CG37, CG45, CG49, CG86) were collected for gene expression analysis. The diameter of the 15th internodes was harvested at 42 days after transplanting. The stems of three plants from each material were mixed into one sample, with three replicates for each material. Total RNA was extracted using an RNeasy Plant Mini Kit (TaKaRa 9769, Takara Bio, Inc., Otsu, Japan). The RNA concentration and quality were examined by electrophoresis on a 1% (*w*/*v*) agarose and NanoDrop One (Thermo Scientific, Waltham, MA, USA). Reverse transcription of the extracted RNA into cDNA was performed with the UEIris Ⅱ RT-PCR System (Biodee, Beijing, China). The quantitative reverse transcription-polymerase chain reaction (qRT-PCR) was performed using SYBR Premix Ex Taq II (TaKaRa Bio, Inc., Otsu, Japan). *Actin* was used as a reference gene for normalizing gene expression values [33]. Specific primers for each gene are listed in Table S2. The 20 µL mixture system of qRT-PCR contained 2 µL of cDNA (50 ng·µL^−1^), 0.4 µL each of forward and reverse primers (10 µmol·L^−1^), 7.2 µL ddH2O, and 10 µL of 2 X ChamQ Universal SYBR qPCR Master Mix (Vazyme, Nanjing, China). The PCR reaction program in the CFX96 Real-Time System (Bio-Rad, Hercules, CA, USA) is 95 ℃ for 30s, 40 cycles (95 ℃ for 10 s, 60 ℃ for 30 s), melt curve 60 ℃ to 95 ℃, and increment 0.5 ℃. Relative gene expressions were calculated using the 2^−∆∆Ct^ method [34].

## 3. Results

### 3.1. Genetic Diversity of Stem Diameter in Core Germplasms

**Figure 1:**
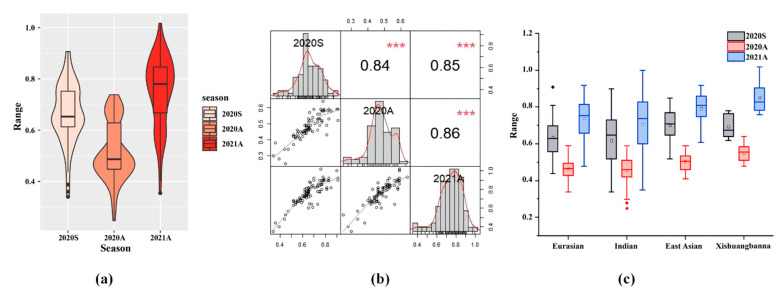
Phenotypic analysis of cucumber stem diameter in three seasons. (**a**) Violin and box plots depicting phenotypic distribution of cucumber stem diameter in 2020S, 2020A, and 2021A. (**b**) Frequency distribution and Spearman rank correlations of the mean values of the CG lines in 2020S, 2020A, and 2021A. *** indicates significance *p* < 0.01. (**c**) Phenotypic distribution of cucumber stem diameter in four ecotypes of the CG lines in 2020S, 2020A, and 2021A. ◆ indicate significance outliers.

The cucumber stem diameters of 88 cucumber CG lines were measured in Spring 2020, Autumn 2020 and Autumn 2021,respectively. The stem diameter in different seasons all showed a continuous normal distribution, which suggests that the cucumber stem diameter is controlled by polygenes. The cucumber stem diameters in three seasons range from 0.34 to 0.91, 0.25 to 0.74 and 0.35 to 1.02, with the mean of 0.66, 0.48 and 0.76, respectively. The overall diameter of cucumber stem in Autumn 2021 was larger, which may be due to the later developmental stage of plants when phenotyped. The coefficients of variation of stem diameter in the three seasons were 17.32%, 15.10% and 16.08%, respectively (Table 1). The correlation of stem diameter in the three seasons was between 0.84 and 0.86, showing a highly significant correlation (*p* < 0.001) (Figure 1a,b). Moreover, the 88 CG lines were divided into four ecotypes [26], including the East Asian type (33 CG lines), the Eurasian type (20 CG lines), the Indian type (19 CG lines), and the Xishuangbanna type (8 CG lines); it was found that the stem of East Asian cucumber was thicker than that of European and Indian cucumber. However, it was difficult to group the Xishuangbanna type cucumber since only eight CG lines were included (Table S3; Figure 1c).

### 3.2. Clustering Analysis of Stem Diameter in Core Germplasms

**Figure 2:**
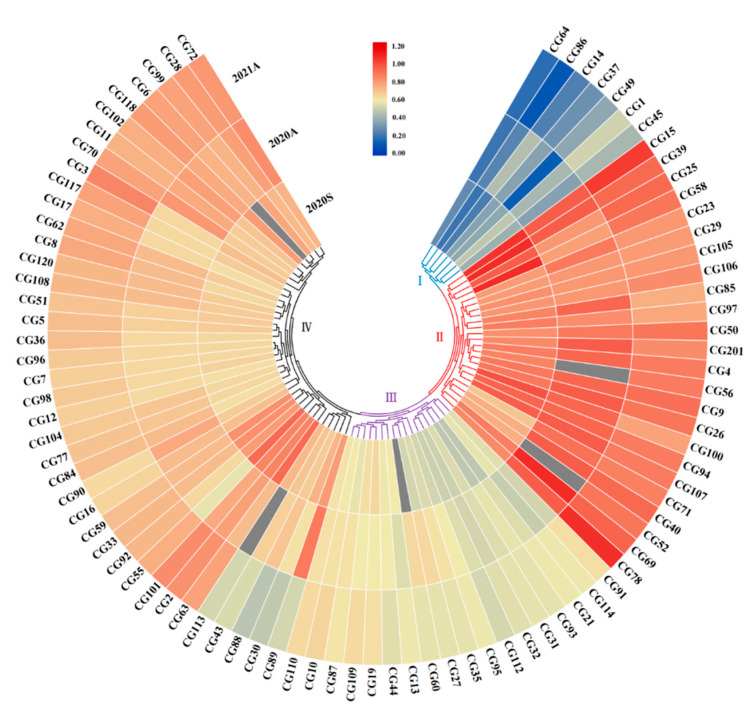
Heatmap depicting the phenotypic distribution of cucumber stem diameter in the three seasons. The four clusters of core germplasm (CG) lines are numbered with Ⅰ to Ⅳ. Color intensity indicates stem thickness of the line. Gray means data were unavailable.

According to the phenotypic data of cucumber stems in the three seasons, these CG lines could be grouped into four clusters—: Ⅰ, Thin; Ⅲ, Intermediate thin; Ⅳ, Intermediate thick; Ⅱ, Thick (Table S4; Figure 2). Each cluster contains different ecotypes. The thin stem group contained the Indian and Eurasian types. The intermediate thin stem group contained the East Asian type, the Eurasian type and the Indian type. The thick stem group and intermediate thick stem group contained four ecotypes, most of which were the East Asian type. Here, the CG lines with the stem diameter falling within the top 20% in all three seasons were considered thick stem materials, while those within the top 20% in all three seasons were considered thin stem materials. Finally, 15 thick stem lines and 13 thin stem lines were selected and used for further analysis (Table S5).

### 3.3. Genome-Wide Association Study of Stem Diameter in Core Germplasms

**Figure 3:**
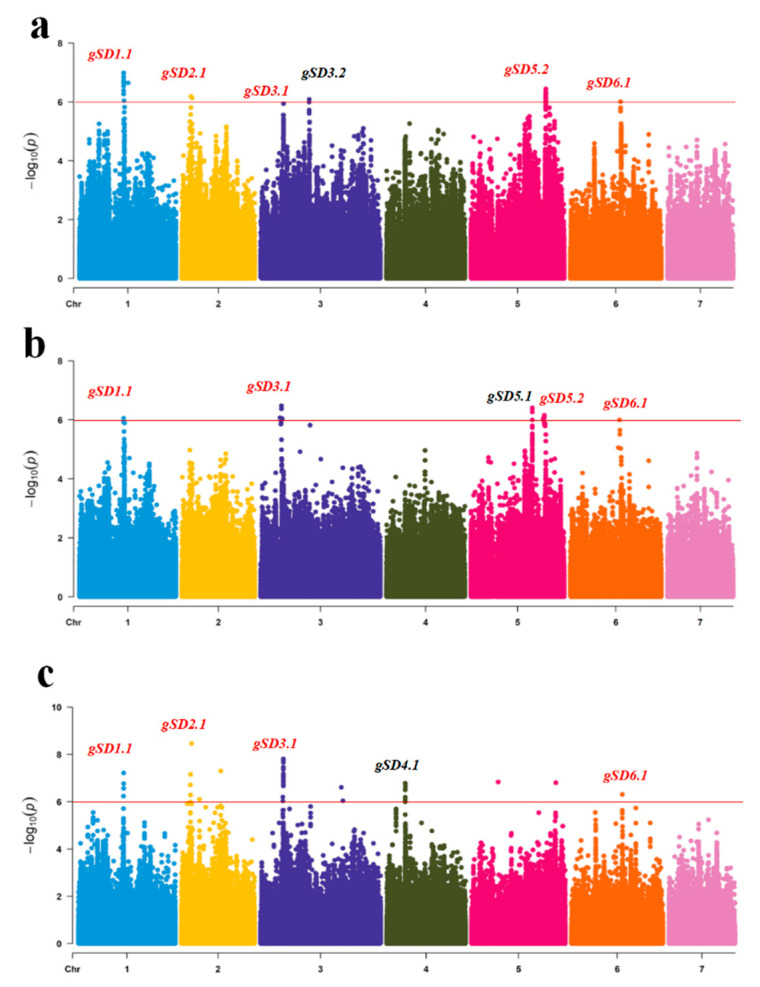
Genome-wide association study (GWAS) analysis of cucumber stem diameter in (**a**) 2020S; (**b**) 2020A; (**c**) 2021A. Manhattan plots of the cucumber stem diameter in three seasons. The red line represents a significant threshold of GWAS (−log_10_*p*)= 6). The red font indicates repeatedly detected loci.

The 88 CG lines were used for GWAS analysis using the FaST-LMM model by combining the stem diameter phenotypic data in all three seasons and gene sequence data. With a threshold of six, eight genetic loci related to stem diameter were detected. These eight loci were distributed on seven chromosomes. Among these eight loci, five loci— namely, *gSD1.1* (2020S, 2020A and 2021A), *gSD2.1* (2020S and 2021A), *gSD3.1* (2020S, 2020A and 2021A), *gSD5.2* (2020S and 2020A) and *gSD6.1* (2020S, 2020A and 2021A)—could be detected in two or more seasons, which could be considered as stable loci for cucumber stem diameter (Table 2; Figure 3).

### 3.4. Analysis of Candidate Genes for GWAS Stable Loci of Stem Diameter in Core Germplasms

Based on the results of the association analysis in three seasons, stable loci that were repeatedly detected were selected for further analysis, the 50-kb region was selected for LD decay analysis [24], and all genes were acquired according to the cucumber genome. Fifteen lines with thick stems and thirteen lines with thin stems were used for gene haplotypes and expression analysis. Based on the linkage disequilibrium sequences of the associated SNP loci, 37 genes were selected (Table S7). The candidate genes include the oxygenase superfamily protein, domain-containing protein, calmodulin-binding protein, zinc finger matrin-type protein and kinesin-like protein, as well as those involved in the growth-regulating factor; α-glucan water dikinase and wall-associated receptor kinase.

#### 3.4.1. *gSD1.1* Candidate Gene Analysis

**Figure 4:**
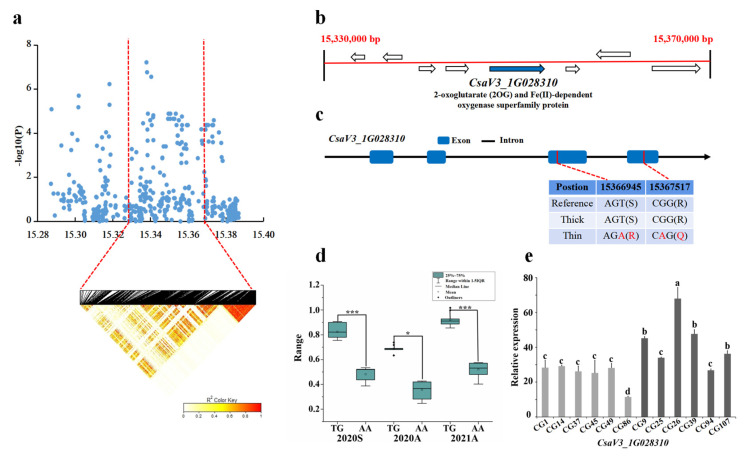
Identification of the stem diameter-related gene at locus *gSD1.1*. (**a**) Manhattan map of near 100 kb (**top**) and LD heatmap (**bottom**) surrounding the peak of *gSD1.1*. (**b**) Eight genes predicted in the LD block region. Blue represents the genes that are different. (**c**) SNP variation of the candidate gene *CsaV3_1G028310* among thick and thin stem lines (Table S6). Two SNPs (in red) were located in CDS, resulting in amino acid changes. (**d**) Haplotype difference of *CsaV3_1G028310*. *** indicatess significance *p* < 0.001. ** indicatess significance *p* < 0.01. * indicatess significance *p* < 0.05. (**e**) Relative expression analysis of *CsaV3_1G028310*. Gray column represents the thin stem samples (CG1, CG14, CG37, CG45, CG49, CG86); Black column represents thick stem samples (CG9, CG25, CG26, CG39, CG94, CG107). a,b,c and d represent the level of significant difference from high to low.

For locus *gSD1.1*, the candidate region (Chr.1: 15,297–15,397 kb) was analyzed by pairwise LD correlations, and we focused on the region from 15,330,000 bp to 15,370,000 bp in the LD block (Figure 4a). Four candidate genes located in this region were *CsaV3_1G028290*, *CsaV3_1G028300*, *CsaV3_1G028310*, and *CsaV3_1G028320* (Figure 4b). Only the SNP on *CsaV3_1G028310* showed significant differences in 15 thick and 13 stable thin stems. A total of 12 SNP mutations occurred in *CsaV3_1G028310*, including the 10th and 12th SNP in the CDS region which resulted in non-synonymous mutations in two amino acids. Among thick and thin stem lines, 11 out of the 15 thick lines carried the GCCGGAACTTGG haplotype, whereas 7 of the 13 thin stem lines carried the ATTATGGACAAA haplotype (Figure 4c,d). Gene annotation showed that *CsaV3_1G028310* is associated with antibiotic biosynthesis. Its homolog in Arabidopsis *AT5G20400* encodes a protein that can recognize mRNAs that move between different organs under nutritionally restrictive conditions, following a phloem-dependent distribution pathway that transports sugars from photosynthetic tissue to stems and roots [35]. On analyzing the gene expression pattern, the expression of *CsaV3_ 1G028310* was lower in the thin stem samples (CG1, CG14, CG37, CG45, CG49, CG86) than in the thick stem samples (CG9, CG25, CG26, CG39, CG94, CG107) (Figure 4e). Therefore, the *CsaV3_1G028310* may be a candidate gene controlling stem development.

#### 3.4.2. *gSD2.1* Candidate Gene Analysis

**Figure 5:**
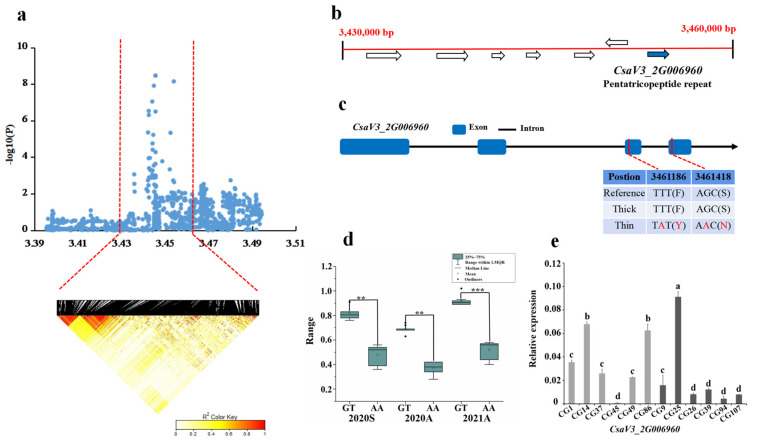
Identification of the stem diameter-related gene at locus *gSD2.1*. (**a**) Manhattan map of near 100 kb (**top**) and LD heatmap (**bottom**) surrounding the peak of *gSD2.1*. (**b**) Seven genes predicted in the LD block region. Blue represents the genes that are different. (**c**) SNP variation of the candidate gene *CsaV3_2G006960* among thick and thin stem lines (Table S6). Two SNPs (in red) were located in CDS, resulting in amino acid changes. (**d**) Haplotype difference of *CsaV3_2G006960*. *** indicatess significance *p* < 0.001. ** indicatess significance *p* < 0.01. * indicatess significance *p* < 0.05. (**e**) Relative expression analysis of *CsaV3_2G006960*. Gray column represents the thin stem samples (CG1, CG14, CG37, CG45, CG49, CG86); Black column represents thick stem samples (CG9, CG25, CG26, CG39, CG94, CG107). a,b,c and d represent the level of significant difference from high to low.

For locus *gSD2.1*, the candidate region (Chr.2: 3400–3490 kb) was analyzed by pairwise LD correlations, and we focused on the region from 3,430,000 bp to 3,460,000 bp in the LD block (Figure 5a). Seven candidate genes located in this region were *CsaV3_2G006900*, *CsaV3_2G006910*, *CsaV3_2G006920*, *CsaV3_2G006930*, *CsaV3_2G006940*, *CsaV3_2G006950*, and *CsaV3_2G006960* (Figure 5b). Only the SNP on *CsaV3_2G006960* showed significant differences in 15 thick and 13 thin stems. Among thick and thin stem lines, 10 out of the 15 thick lines carried the GGCAAGATGCGG haplotype, whereas 7 of the 13 thin stem lines carried the CTTGGAGAAGAA haplotype. The SNPs in the region resulted in two amino acid changes, which were located in CDS (Figure 5c,d). Gene annotation revealed that *CsaV3_2G006960* is pentatricopeptide repeat. Its homolog in Arabidopsis, *AT1G09190*, is a class of superfamily proteins associated with TPR related to mitochondrial RNA processing. Moreover, on analyzing the gene expression pattern, the expression of *CasV3_2G006960* was highest in CG25, and those of the other five thick stem samples (CG9, CG26, CG39, CG94, CG107) were much lower than that of CG25. CG45 had the lowest gene expression compared to the other five thin stem samples (CG1, CG14, CG37, CG49, CG86).

#### 3.4.3. *gSD3.1* Candidate Gene Analysis

**Figure 6:**
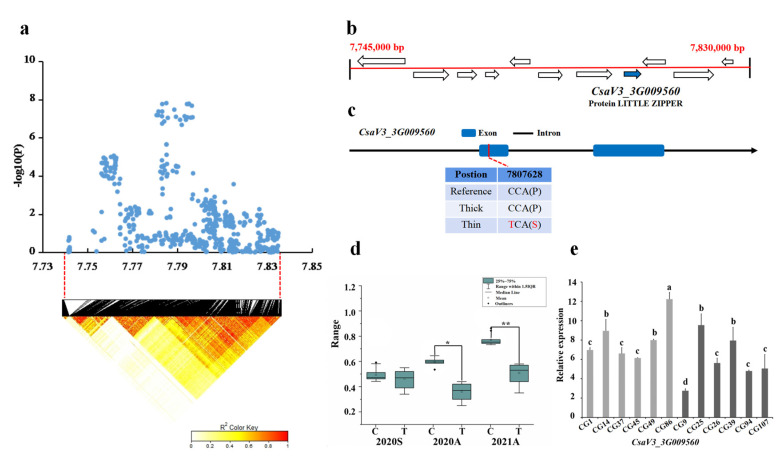
Identification of the stem diameter-related gene at locus *gSD3.1*. (**a**) Manhattan map of near 100 kb (**top**) and LD heatmap (**bottom**) surrounding the peak of *gSD3.1*. (**b**) Eleven genes predicted in the LD block region. Blue represents the genes that are different. (**c**) SNP variation of the candidate gene *CsaV3_3G009560* among thick and thin stem lines (Table S6). One SNP (in red) was located in CDS, resulting in amino acid changes. (**d**) Haplotype difference of *CsaV3_3G009560*. *** indicatess significance *p* < 0.001. ** indicatess significance *p* < 0.01. * indicatess significance *p* < 0.05. (**e**) Relative expression analysis of *CsaV3_3G009560*. Gray column represents the thin stem samples (CG1, CG14, CG37, CG45, CG49, CG86); Black column represents thick stem samples (CG9, CG25, CG26, CG39, CG94, CG107). a,b,c and d represent the level of significant difference from high to low.

For locus *gSD3.1*, a candidate region (chr3: 7740–7830 kb) was analyzed by LD correlation analysis, and we focused on the region from 7,745,000–7,830,000 bp in the LD block; 11 candidate genes were located in this region (Figure 6a). Only the SNP on *CsaV3_3G009560* showed significant differences between 15 thick and 13 thin stems haplotypes in Autumn 2020 and Autumn 2021 (Figure 6b). A total of seven SNP occurred in *CsaV3_3G009560*, including the 6th SNP in the CDS region, resulting in non-synonymous mutations in one amino acid. Among thick and thin stem lines, 9 out of the 15 thick lines carried the TTGAGCC haplotype, whereas 11 of the 13 thin stem lines carried the CAATATA haplotype (Figure 6c,d). Gene annotation showed that *CsaV3_3G009560* is a LITTLE ZIPPER protein. Its homolog in Arabidopsis *AT2G45450* interacts with the HD-Zip protein to form a heterodimer, which directly affects the activity of stem tip tissue and the abnormality of stem cells [36]. According to gene expression pattern analysis, the expression of *CsaV3_3G009560* was higher in thin stem samples (CG1, CG14, CG37, CG45, CG49, CG86) than that of the thick stem samples (CG9, CG26, CG94, CG107) (Figure 6e).

#### 3.4.4. *gSD5.2* Candidate Gene Analysis

**Figure 7:**
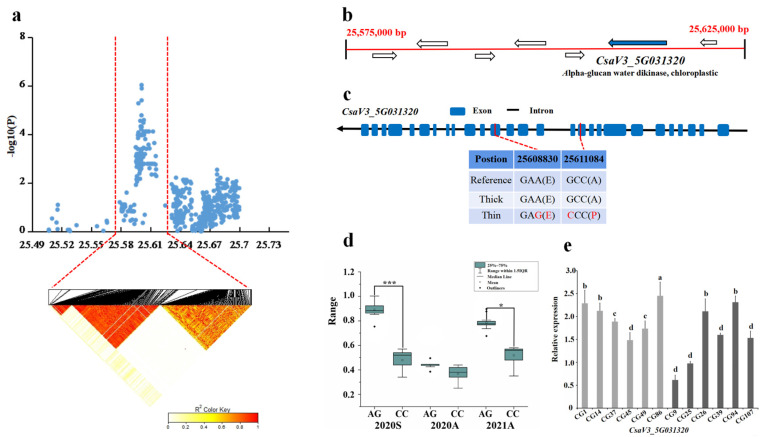
Identification of the stem diameter-related gene at locus *gSD5.2*. (**a**) Manhattan map of near 300 kb (**top**) and LD heatmap (**bottom**) surrounding the peak of *gSD5.2*. (**b**) Seven genes predicted in the LD block region. Blue represents the genes that are different. (**c**) SNP variation of the candidate gene *CsaV3_5G031320* among thick and thin stem lines (Table S6). Two SNP (in red) were located in CDS, resulting in amino acid changes. (**d**) Haplotype difference of *CsaV3_5G031320*. *** indicatess significance *p* < 0.001. ** indicatess significance *p* < 0.01. * indicatess significance *p* < 0.05. (**e**) Relative expression analysis of *CsaV3_5G031320*. Gray column represents the thin stem samples (CG1, CG14, CG37, CG45, CG49, CG86); Black column represents thick stem samples (CG9, CG25, CG26, CG39, CG94, CG107). a,b,c and d represent the level of significant difference from high to low.

For locus *gSD5.2*, the candidate region (Chr5: 25,490–25,700 kb) was analyzed by pairwise LD correlation, and we focused on a region from 25,575,000–25,625,000 bp in the LD block (Figure 7a). Seven candidate genes were located in this region, *CsaV3_5G031270*, CsaV3_5G031290, *CsaV3_5G031300*, *CsaV3_5G031310*, *CsaV3_5G031320*, and *CsaV3_5G031330* (Figure 7b). In *CsaV3_5G031320*, 21 SNPs were significantly different in 15 thick stem lines and 13 thin stem lines haplotypes. Of these 21 SNPs, the 9th and 15th SNP in the CDs region resulted in non-synonymous mutations that led to amino acid changes (Figure 7c). Among the thick and thin stem lines, 13 out of the 15 thick stem lines carried the TCATTCCAAGGAAGCAAATAG haplotype, and all the thin stem lines carried the CAGGAATCGATGGCTGGTCTA haplotype (Figure 7d). The gene *CsaV3_5G031320* encoded a kinase function in chloroplast. Its homolog Arabidopsis *AT1G10760* encodes the dextran and water dikinase required for starch degradation [37]. On analyzing the gene expression pattern, the expression *CsaV3_5G031320* was significantly higher in thin stem CG86 sample compared to in the other stem samples, and it was the lowest in the thick stem CG9 sample. Overall, this gene is expressed higher in thin stem samples than thick stem samples, thus the *CsaV3_5G031320* gene may be a candidate controlling the *gSD5.2* locus (Figure 7e).

#### 3.4.5. *gSD6.1* Candidate Gene Analysis

**Figure 8:**
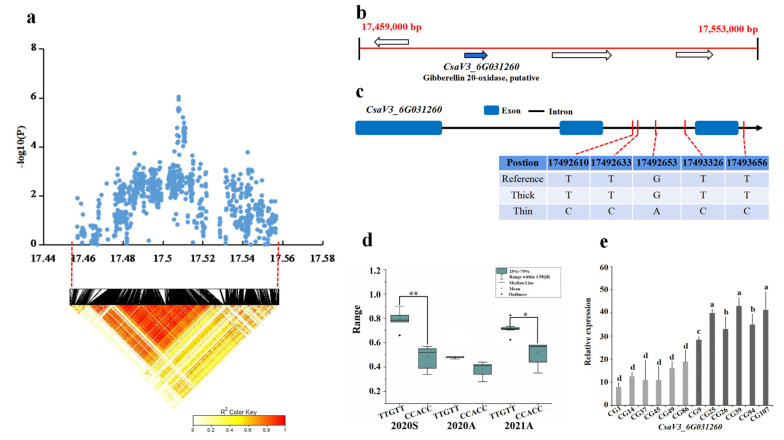
Identification of the stem diameter-related gene at locus *gSD6.1*. (**a**) Manhattan map of near 100 kb (**top**) and LD heatmap (**bottom**) surrounding the peak of *gSD6.1*. (**b**) Four genes predicted in the LD block region. Blue represents the genes that are different. (**c**) SNP variation of the candidate gene *CsaV3_6G031260* among thick and thin stem lines (Table S6). (**d**) Haplotype difference of *CsaV3_6G031260*. *** indicatess significance *p* < 0.001. ** indicatess significance *p* < 0.01. * indicatess significance *p* < 0.05. (**e**) Relative expression analysis of *CsaV3_6G031260*. Gray column represents the thin stem samples (CG1, CG14, CG37, CG45, CG49, CG86); Black column represents thick stem samples (CG9, CG25, CG26, CG39, CG94, CG107). a,b,c and d represent the level of significant difference from high to low.

For locus *gSD6.1*, the candidate region (Chr6: 17,459–17,553kb) was analyzed by pairwise LD correlation; we focused on the region from 17,459,000 bp to 17,553,000 bp in the LD block (Figure 8a). Four genes were predicted in this region, namely *CsaV3_6G031250*, *CsaV3_6G031260*, *CsaV3_6G031270*, and *CsaV3_6G031280*, three of which encoded a cell wall-associated receptor kinase protein, while *CsaV3_6G031260* was associated with gibberellin (Figure 8b). Only the SNP on *CsaV3_6G031260* showed significant differences in 15 thick and 13 thin stem lines haplotypes (2020S and 2021A). Five SNPs were found in the intron, and all of them have synonymous mutations. Among thick and thin stem lines, 8 out of the 15 thick stem lines carried the TTGTT haplotype, whereas 11 out of the 13 thin stem lines carried the CCACC haplotype (Figure 8c,d). Its homolog in Arabidopsis, *AT5G51810*, is involved in gibberellin synthesis and is upregulated in plant tissues. On analyzing the gene expression pattern, the expression of *CsaV3_6G031260* in these six thin stem samples (CG1, CG14, CG37, CG45, CG49, CG86) was lower than that in thick stem samples (CG9, CG25, CG26, CG39, CG94, CG107) (Figure 8e). Thus, the *CsaV3_6G031260* gene is a candidate to control the *gSD6.1* locus.

## 4. Discussion

Stem formation is the result of the activity of apical stem terminal. Cells in the outer layer of the stem terminal meristem participate in the formation of the primordium and lateral bud meristem of lateral organs, while the internal cell population forms the main stem through division and differentiation. The activity of the meristem not only promotes the occurrence of lateral organs, but its initial developmental state will affect the diameter of the plant stem. Stem native xylem vascular lignification occurs in the early stages of vascular bundle differentiation, and the stem vascular bundle, as a major part of the transport of plant matter, plays an important role in the transport and distribution of water, minerals, sugars and amino acids during plant growth [38]. Tissue anatomical observation showed that the mature stem of cucumber was a primary structure, composed of epidermis, cortex and vascular bundle, and cell division dominated by early development of cucumber stem. Huang found that in cucumber varieties with well-developed cortex and a large number of vascular bundles, organic matter runs more and runs faster [39]. The stem thickness of cucumber significantly affects plant growth, water intake, and nutrient absorption. Hu Deyong treated cucumber plants with aerobic irrigation, and found that the accumulation of dry matter in cucumber stems increased, resulting in thicker stem, and significantly increased yield [40]. Studies showed that thick stems will directly increase cucumber yield [41,42]. In this study, cucumber samples from East Asia, Europe, the USA, Xishuangbanna, and India were selected from more than 3000 germplasms all over the world, representing more than 75% of the genetic variation in cucumber [24]. By comparing the geographic origin and ecotypes of various germplasms, the core germplasm was clustered into four groups, of which the thick germplasm includes all four ecological types, and in the thin core germplasm, only the Indian and Eurasian types are included. Of the intermediate thin core germplasm, more than half are East Asian types. After a long period of natural selection and domestication, East Asian types with thick stem samples might be used for breeding and the selection of new varieties. Most germplasms with intermediate thick core germplasm were European greenhouse types. The germplasm with thick stem screened in this study could be used for further gene cloning and genetic breeding.

With the completion of cucumber genome sequencing, it is more efficient to conduct GWAS for identifying genes underlying cucumber complex traits. The method of QTL mapping can only enable the analysis of gene effects varying between parental materials of isolated populations and cannot extensively mine genes involved in stem diameter regulation genome-wide. GWAS studies on cucumber stem diameter have not been reported yet. Here, 88 cucumber CGs were used for GWAS and eight significantly associated loci for stem diameter were detected, *gSD1.1*, *gSD2.1*, *gSD3.1*, *gSD3.2*, *gSD4.1*, *gSD5.1*, *gSD5.2* and *gSD6.1*. There is only one QTL for cucumber stem diameter that has been reported. Li [10] detected two QTLs related to cucumber stem diameter, *qMSD-1* (Chr.1: 630,399 bp) on chromosome 1 between markers OP-V20 and CSFR12, and *qMSD-7* (Chr.7: 20,536,944 bp) on chromosome 7 between markers e23M15a and e23M15b. The QTL on Chr.1, *qMSD-1,* was far from the *gSD1.1* locus (physical position 15,337,811 bp) detected on Chr.1 in this study, suggesting that the eight loci we detected are all novel loci.

Within the stable loci that were detected in more than two seasons, candidate genes for cucumber stem thickness were analyzed. By further investigating the five loci via analyzing *Arabidopsis* homologous genes and haplotypes, stem diameter-related genes within each loci were predicted; they are *CsaV3_1G028310* of *gSD1.1*, *CsaV3_2G006960* of *gSD2.1*, *CsaV3_3G009560* of *gSD3.1*, *CsaV3_5G031320* of *gSD5.2*, and *CsaV3_6G031260* of *gSD6.1*. Homologous of *CsaV3_1G028310* in Arabidopsis is *AT5G20400*, which encodes a promoting protein synthesis. It promotes cellulase synthesis at the base of the Arabidopsis stem, causing stem changes [43]. Zhang [44] cloned a gene that controls stem strength of maize stiff1, which promotes the synthesis of the F-box protein. They found that a 27.2 kb transposon element inserted into the stiff1 gene promoter region inhibited the transcription of stiff1 gene, resulting in increased content of cellulose and lignin in cell wall and enhanced stem strength. For the *gSD2.1* locus, *CsaV3_2G006960* was a pentapeptide repeat protein, and its Arabidopsis homologous gene is a superfamily protein, and the relationship with the stem structure needs to be further determined. For the *gSD3.1* locus, *CsaV3_3G009560* was related to the LITTLE ZIPPER protein. The Arabidopsis homologous gene is *AT2G45450*. According to Kim [36], *AT2G45450* is related to the shoot apical meristem in Arabidopsis. Mayer found that the *Arabidopsis* gene *WUS* plays an important role in maintaining the structure and function of stem-end meristem tissue, which encodes the same family protein as the gene *AT2G45450*, which is expressed only in the central layer cells of stem-end meristem tissue, which can maintain the ability of stem-end meristem cells to divide and differentiate. The mutation of this gene will improve the cell division capacity of stem-end meristem, increase cell number, and expand the stem [45]. Tu [46] conducted QTL mapping analysis and genetic complementarity test of lodging resistance and found that the candidate gene osckx2/gn1a was related to cytokinin oxidase produced by stem tip and affects the development of stem. Therefore, *CsaV3_3G009560* in cucumber may regulate stem thickness by influencing shoot apical cell division. *CsaV3_5G031320* gene expression was significantly different among thick and thin stem samples. Its homolog in Arabidopsis is involved in CO_2_-induced metabolic changes [47], but whether it is related to the development of plant stems has not been reported. *CsaV3_5G031320* may control cucumber stem thickness, but the regulatory mechanism remains unclear. For the *gSD6.1* locus, *CsaV3_6G031270* only had haplotype changes in the intron region and did not lead to amino acid changes, but its expression was significantly different among thick and thin stem samples. In Arabidopsis, *AT1G21230*, the homolog of *CsaV3_6G031270*, is involved in metabolite transport; however, its function in cucumber remains unclear. In addition, *CsaV3_2G006930* within *gSD2.1* encodes an calmodulin-binding protein. Wang [48] found that *FUL2* was mainly expressed in stem, its overexpression led to thinning stem in tomato plants, and the expression of calmodulin-binding protein *SIEXP1* was downregulated. Therefore, it is possible that *CsaV3_2G006930* interacts with other genes to regulate cucumber stem growth. The function of these candidate genes and their mechanism in regulation stem development could be further studied.

## 5. Conclusions

We screened 15 thick stem materials that could be used for breeding cucumber with thick stem. Additionally, a total of eight loci related to stem diameter were identified by GWAS analysis, among which five robust loci were repeatedly detected. Five candidate genes related to cucumber stem diameter were predicted. The results of this study have important application value for breeding new cucumber varieties with strong growth potential and high yield, and lay a foundation for the cloning and molecular mechanism research of cucumber stem diameter-related genes.

## Figures and Tables

**Table 1:** Statistical analysis of the cucumber stem diameter.

Season	Maximum	Minimum	Mean ± SD	Kurtosis	Skewness	(%)CV	*p* Value
2020S	0.91	0.34	0.67 ± 0.12	0.41	−0.39	17.32	0.07 > 0.05
2020A	0.74	0.25	0.51 ± 0.11	0.97	−0.61	15.10	0.10 > 0.05
2021A	1.02	0.35	0.76 ± 0.14	0.11	−0.86	16.08	0.20 > 0.05

2020S: In the Spring of 2020; 2020A: In the Autumn of 2020; 2021A: In the Autumn of 2021.

**Table 2:** GWAS signal sites and corresponding SNP markers of cucumber stem diameter in three seasons.

Season	Signal Sites	SNP	Chr.	Physical Position (bp)	−log_10_ *p* Value
2020S	*gSD1.1*	S1_15337811	1	15,337,811	6.95
	*gSD2.1*	S2_3445551	2	3,445,551	6.13
	*gSD3.1*	S3_7785079	3	7,785,079	6.01
	*gSD3.2*	S3_16649668	3	16,649,668	6.11
	*gSD5.2*	S5_25600905	5	25,600,905	6.44
	*gSD6.1*	S6_17507945	6	17,507,945	6.03
2020A	*gSD1.1*	S1_15337811	1	15,337,811	6.05
	*gSD3.1*	S3_7785079	3	7,785,079	6.24
	*gSD5.1*	S5_20992082	5	20,992,082	6.41
	*gSD5.2*	S5_25600905	5	25,600,905	6.03
	*gSD6.1*	S6_17507945	6	17,507,945	6.05
2021A	*gSD1.1*	S1_15337811	1	15,337,811	7.42
	*gSD2.1*	S2_3445551	2	3,445,551	8.46
	*gSD3.1*	S3_7785079	3	7,785,079	7.81
	*gSD4.1*	S4_6318227	4	6,318,227	6.78
	*gSD6.1*	S6_17507945	6	17,507,945	6.13

## Data Availability

All relevant data are within this article and its Supplementary Data file.

## Supplementary Materials

The following supporting information can be downloaded at: https://www.mdpi.com/article/10.3390/genes13061095/s1, Table S1: Code and Source of Core Germplasms. Table S2: Oligos used for genes qRT-PCR. Table S3: Depicting phenotypic distribution of cucumber stem diameter in four ecotypes of the CG lines. Table S4: Cluster analysis of core germplasm stem diameter. Table S5: The selected thin stem samples (blue block) and thick stem samples (pink block). Table S6: Hyplotypes of SNPs in the candate genes of each locus. Table S7: Candidate genes and gene annotations.
